# Merkel Cell Carcinoma with Distant Metastasis to the Clivus Causing Symptoms Mimicking Tolosa–Hunt Syndrome: A Case Report and Literature Review

**DOI:** 10.3389/fneur.2017.00409

**Published:** 2017-08-18

**Authors:** Kwo Wei David Ho, Peter A. Drew, Miguel Chuquilin

**Affiliations:** ^1^Department of Neurology, University of Florida, Gainesville, FL, United States; ^2^Department of Pathology, University of Florida, Gainesville, FL, United States

**Keywords:** Merkel cell carcinoma, clivus, metastasis, Tolosa–Hunt syndrome, cranial nerve palsies, cavernous sinus

## Abstract

Merkel cell carcinoma (MCC) is an uncommon but highly malignant neuroendocrine tumor of the skin. MCC can metastasize, but involvement of the central nervous system is rare. Here, we report a case of rapidly progressing metastatic MCC to the clivus and bilateral cavernous sinus in an immunocompromised patient. This case is unique in that it is the first case report showing MCC metastasis to the clivus from a distant site. It also demonstrates that a MCC metastasis can masquerade with symptoms of Tolosa–Hunt syndrome. A literature review on MCC with CNS metastasis is presented.

## Introduction

A 60-year-old man presented to the emergency room with right eyelid ptosis, diplopia, and pain behind the right eye. Symptoms started 3 weeks ago with pain behind the right eye that was sharp and intermittent in nature, and each time lasting a few hours. He saw his ophthalmologist 1 week after symptom onset and was told to keep the eye moist with eye drops. However, the right retroocular pain gradually worsened and became 4/10 in intensity. During this time, he also developed right-sided ptosis and diplopia with associated nausea and vomiting.

His medical history was significant for diabetes mellitus, end stage renal disease status post combined pancreatic and renal transplant 8 years ago (on tacrolimus, prednisone, and mycophenolate), coronary artery disease and left shoulder Merkel cell carcinoma (MCC) (T2 N1 M0 stage IIIB) with metastasis to the left axilla that was resected 4 months prior to his presentation. At the time of his initial diagnosis of MCC, patient refused chemotherapy because of the risk of transplant rejection and he only received local radiotherapy. In terms of surgical history, he had right cataract surgery 1 year ago.

His family history was significant for father with a stroke and mother with hypertension and renal disease. He never smoked and drank three glasses of wine per week. He did not use illicit drugs. He was a retired laboratory technician at a particle board plant. His home medications included amlodipine, aspirin, carvedilol, cholecalciferol, cyanocobalamin, doxazosin, magnesium oxide, mycophenolate, tacrolimus, albuterol, amoxicillin, atorvastatin, cetirizine, clonidine, clopidogrel, furosemide, levothyroxine, lisinopril, mometasone, multiple vitamin, prednisone, and ranitidine.

On physical exam, vital signs were BP 122/63, pulse 65, respiratory rate 14, SpO_2_ 96%.

**Figure 1:**
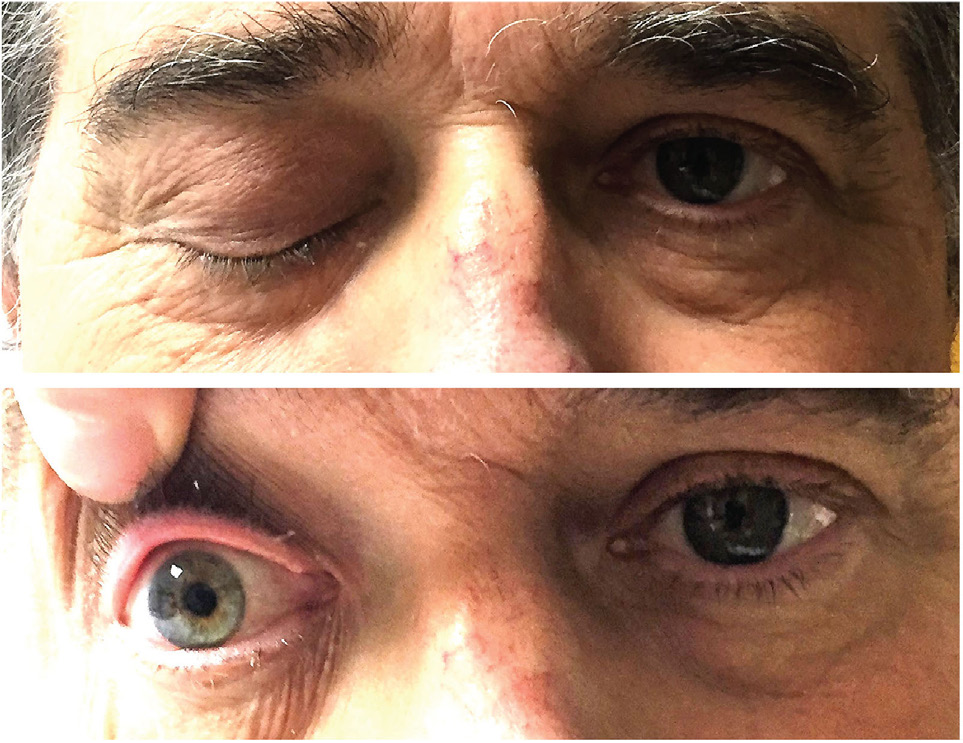
Ptosis of the right eye and fixed and dilated right pupil.

There was complete right-sided ptosis (he was unable to open it), with complete right eye ophthalmoplegia. The right pupil was dilated, 7 mm in size and fixed. Shining light to the right pupil constricted the left pupil. Left pupil was 3 mm in size and reactive to light, but shining light into the left pupil did not constrict the right pupil. The left eye had complete abduction paralysis (Figure 1; Video S1 in Supplementary Material). Visual acuity was 20/40 OD, 20/20 OS. Intraocular pressures were normal. Proptosis was not present. Dilated fundus exam showed temporal pallor of the disk in the right eye, and slight pallor of the disk in the left eye. Pinprick was mildly impaired in the right V1 and left V3 distributions. The rest of the neurological exam was unremarkable.

**Figure 2:**
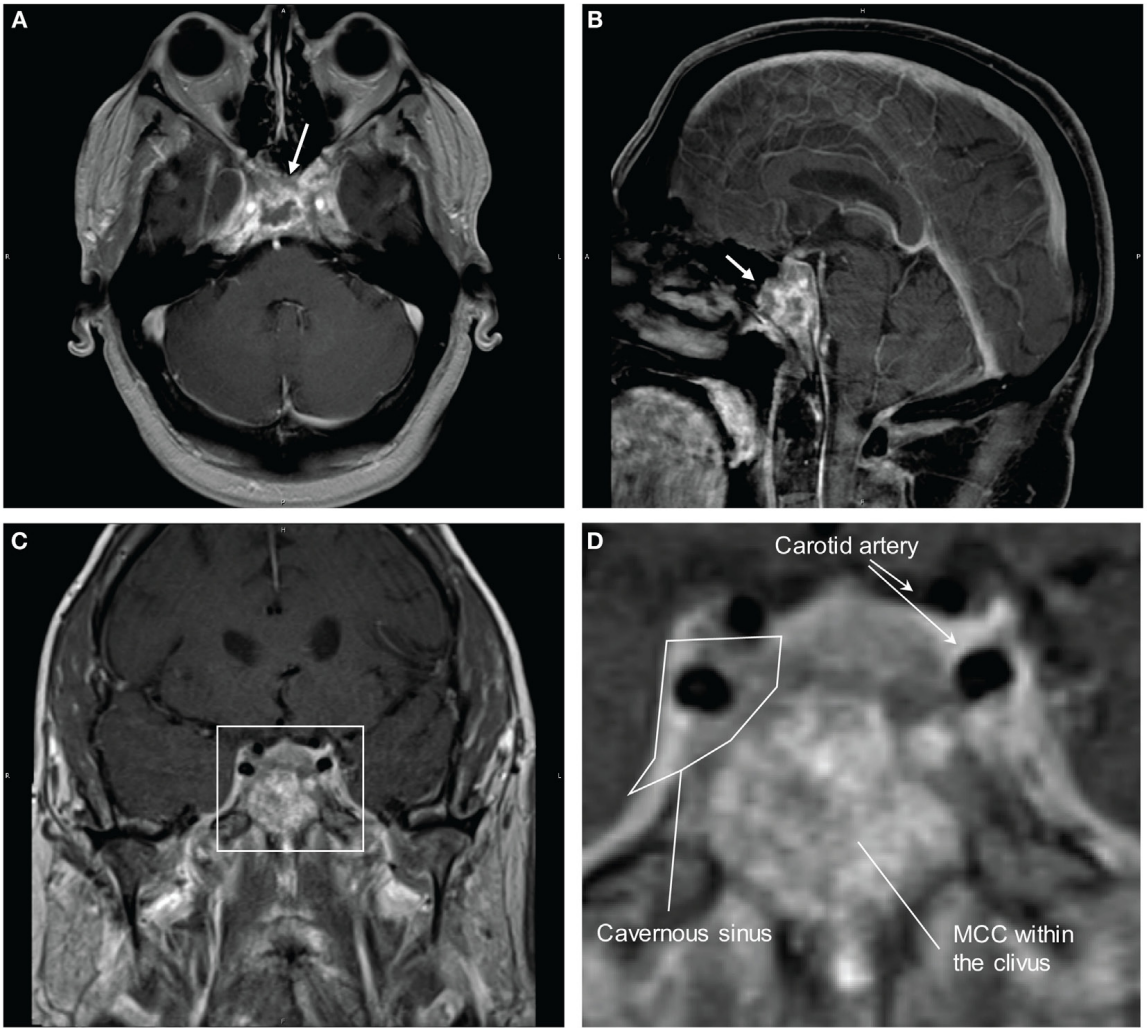
MRI brain with contrast showing Merkel cell carcinoma (MCC) metastasis to the clivus and invasion into the cavernous sinus. **(A)** Transverse view. Arrow is pointing to the mass in the clivus. **(B)** Sagittal view. Arrow is pointing to the mass in the clivus. **(C)** Coronal view. **(D)** Magnified view of the cavernous sinus from **(C)**. There is invasion of the metastatic MCC into the cavernous sinus with likely impingement on the cranial nerves.

**Figure 3:**
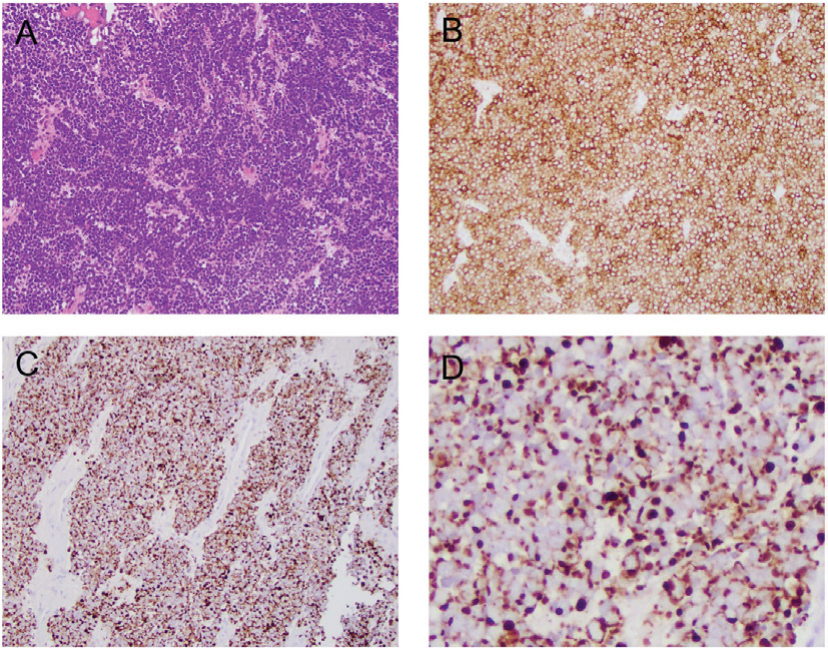
Histological and immunophenotypical features of the clival mass. **(A)** H&E stain at 20× showing small round blue cells. **(B)** CD56 stain positive. **(C)** Cytokeratin CAM 5.2 stain positive. **(D)** Magnified view of the cytokeratin CAM 5.2 stain showing perinuclear dot-like pattern.

Brain MRI with contrast 4 months prior to his presentation did not reveal any abnormality. Differential diagnosis on admission included multiple cranial neuropathies secondary to Tolosa–Hunt syndrome, cavernous sinus thrombosis, infection, granulomatous process or neoplasm, especially in the setting of MCC. Brain MRI showed infiltrative, peripherally enhancing tumor centered within the clivus with soft tissue extension into the cavernous sinus (Figure 2). Brain MRV was performed without contrast due to concern for his transplanted kidney function and the cavernous sinus was not well visualized. CT of the chest showed left axillary and left low cervical lymphadenopathy but negative for lung masses. He was started on Dexamethasone 4 mg q6 hours to reduce tumor inflammation. Biopsy of the clival mass was performed with stereotactic, stealth CT-guided bilateral endoscopic sphenoidotomy and partial ethmoidectomy. The posterior vomer and intersinus septum were removed within the sphenoid and biopsies were taken from the sphenoid mass seen along the floor. H&E slides of the biopsy showed a small round blue cell tumor. Immunuohistochemical slides showed the tumor was positive for CD56, cytokeratin CAM 5.2 (perinuclear dot-like pattern), and cytokeratin 20 (perinuclear dot-like pattern). The tumor was negative for TTF-1, leukocyte common antigen (LCA), S-100, and melan-A (Figure 3). These findings were consistent with metastatic MCC. Further culture revealed colonization with Scopulariopsis species for which he was started on Posaconazole. He was started on palliative radiation therapy and he declined chemotherapy. Repeat MRI in 1 month and CT scan 2 months later did not reveal progression of the clival metastasis, but new metastatic lesions were found in the cervical spine involving C2, C3, C5, and C6 vertebral bodies. He was put on a cervical collar, but no further surgical or radiation therapy was performed as the patient was asymptomatic. Hospice was considered but the patient declined due to the need to stop his antirejection medications. At the time of submission of this manuscript, 6 months after the initial discovery of the Merkel cell metastasis to the clivus, the patient remained alive.

## Background

Merkel cell carcinoma is a rare but aggressive cutaneous neuroendocrine tumor. Our case is unique in that the patient presented with right III, IV, V1, VI and left V3, VI cranial nerve palsies without proptosis, mimicking Tolosa–Hunt syndrome or bilateral cavernous sinus thrombosis. This is the first case showing distant MCC metastasis to the clivus with invasion of the cavernous sinuses. His presentation with complete right ptosis, fixed and dilated pupil along with impairment of all extraocular movement of the right eye is unique. The only case report of metastatic MCC involving the cavernous sinus was by Chang et al. (1). However, that case presented only with retro-orbital pain and hemifacial, hemi-tongue numbness. In addition, the primary site of the MCC was on the face 2 years prior to his presentation, with subsequent perineural spread to the brain and leptomeningeal dissemination.

Clivus is a part of the cranium at the skull base sitting adjacent to the sphenoid sinuses and cavernous sinuses. The abducens nerves tracks along the clivus and a tumor within the clival space can often cause abducens nerve palsies (2). This patient presented with bilateral abducens nerve palsy, which is compatible with the clival metastasis. The infiltration into the cavernous sinuses caused right CN III, IV, V1. The left V3 nerve can be secondary to the clival mass or to extension of the cavernous sinus tumor infiltration since V3 is located adjacent to both structures (Figure 2D).

Merkel cell carcinoma brain metastases typically occur after a mean period of 19.7 months (3). The patient presented here was diagnosed and treated for MCC just 4 months prior to his presentation. The growth of his MCC metastasis was also rapid. Brain MRI with contrast 3 months prior to his ocular symptoms did not show any sign of metastasis. He progressed rapidly within 3 weeks of symptom onset and his clinical picture was not concerning enough for an ophthalmologist who examined him 2 weeks prior to his presentation.

Our literature review revealed a total of 40 cases of MCC metastasis to the central nervous system (Table 1). The average age at the time of metastasis is 63.66 years old with 60% of the cases being male. 97% of the cases (39 out of 40 cases) had metastasis to the brain or leptomeninges, with the only exception being the report by Chang et al. with perineural spread to the bilateral acoustic meatus and trigeminal cistern (1). The average time for CNS spread after the primary diagnosis is 24.77 months. The survival rate after the CNS diagnosis is 73% at 6 months and 33% at 1 year. This is consistent with the previous report of a median survival time of 8.5 months after a diagnosis of Merkel cell CNS metastasis (4). None of the reported cases described prior immunosuppression before MCC was detected. The reported case here was diagnosed at age of 60, 4 months after the diagnosis of the primary tumor. He had multiple comorbidities including multiorgan transplant status which, along with metastatic MCC, indicate a poor prognosis.

**Table 1:** Literature review of central nervous system metastasis of Merkel cell carcinoma.

Case	Age/sex	Primary lesion	Location of metastasis	Time to CNS disease after primary Dx	Survival after CNS metastasis	Immunosuppression before primary lesion
Kroll et al. (5)	48M	NA	Brain	NA	NA	None
Kroll et al. (5)	70M	NA	Brain	NA	NA	None
Kroll et al. (5)	72F	NA	Meninges	NA	NA	None
Wick et al. (6)	62M	Face	Brain	12 months	1 month	None
Wick et al. (6)	76M	Right retroauricular area	Brain	3 years	0 month	None
Goepfert et al. (7)	NA	NA	Brain	NA	NA	None
Giannone et al. (8)	57F	Scalp	Right frontoparietal	2 months	4 months, survived	None
Grosh et al. (9)	57F	NA	Brain	2 months	12 months	None
Grosh et al. (9)	56M	NA	Brain	≥21 months	NA	None
Hitchcock et al. (10)	52M	Left breast	Brain	3 months	9 months	None
Knox et al. (11)	75F	Right neck	Left cerebellum	4 years	NA	None
Knox et al. (11)	60M	Inguinal nodes	Leptomeninges	10 months	14 months	None
Dudley et al. (12)	64M	NA	Leptomeninges	17 months	6 days	None
Alexander et al. (13)	56M	Left face	Right parietal	Present at dx	3 years	None
Wojak and Murali (14)	78F	NA	Calvaria, Dura	Present at dx	≥1 year	None
Manome et al. (15)	57F	NA	Frontal skull base, brain	Present at dx	NA	None
Small et al. (16)	56M	NA	Brain	Present at dx	22 months	None
Yiengpruksawan et al. (17)	NA	NA	Brain	NA	NA	None
Sharma et al. (18)	57M		Brain	Present at dx	13 months	None
Snodgrass et al. (19)	61M	Forehead	Right parietal, leptomeninges	1 year	6 months	None
Eftekhari et al. (20)	NA	NA	Brain	NA	NA	None
Eftekhari et al. (20)	NA	NA	Brain	NA	NA	None
Eftekhari et al. (20)	NA	NA	Brain	NA	NA	None
Matula et al. (21)	47M	NA	Anterior skull base, dura, brain	Present at dx	12 months	None
Straka et al. (22)	71F	NA	Brain	10 months	2 months	None
Litofsky et al. (23)	86F	External auditory canal	Brain	16 months	≥8 months	None
Ikawa et al. (24)	48F	Left elbow	Right cerebellum	5 years	11 months	None
Eggers et al. (25)	69M	NA	Pons and midbrain, leptomeninges	17 months	<2 months	None
Barkdull et al. (26)	55M	Scalp	Calvaria, Dura	Present at dx	10 months	None
Faye et al. (27)	85M	NA	Right parietal	1 year	NA	None
Faye et al. (27)	68M	NA	Brain, leptomeninges	1 year	7 months	None
Chang et al. (1)	45M	Left temporal skin	Bilateral internal acoustic meatus, left trigeminal cistern	2 years	≥3 months	None
De Cicco et al. (28)	69M	NA	Brain	22 months	6 months	None
Feletti et al. (3)	65F	NA	Pituitary	3.5 years	≥8 months	None
Bailey et al. (4)	75F	Nasal ala	Right parietal	1 year	7 months	None
Bailey et al. (4)	77F	Left scalp	Parietal lobe, scalp	Present at dx	≥16 months	None
Bailey et al. (4)	51F	Right calf/inguinal lymph nodes	Right temporal lobe	4 years	>21 months, survived	None
Abul-Kasim et al. (29)	65M	Left inguinal, iliac, aortocaval lymph nodes	Right parietal, lumbar spine	NA	8 months, survived	None
Seaman et al. (30)	78M	Right groin	Left central pontine angle mass	2 months	5 months, survived	None
This report	60M	Left shoulder	Clivus, cavernous sinus	4 months	–	Yes
Average	63.66 years old	–	–	24.76 ± 19.5 months	6 months survival: 73%	
	60% M				12 months survival: 33%	

## Discussion

When the patient first presented, Tolosa–Hunt syndrome and cavernous sinus thrombosis were the top two differential diagnoses. However, cavernous sinus thrombosis typically presents with proptosis (80–100%) (31), and our patient did not, making Tolosa–Hunt syndrome a more compelling etiology on presentation. Tolosa–Hunt syndrome is an idiopathic, sterile inflammation affecting the cavernous sinus. It is defined by the International Headache Society as “episodic orbital pain associated with paralysis of one or more of the third, fourth, and/or sixth cranial nerves, which usually resolves spontaneously but tends to relapse and remit” (32). Tolosa–Hunt syndrome is typically unilateral, with bilateral symptoms occurring in only about 4% of the cases (33). Cranial nerve III is the most commonly affected cranial nerve (78.3%), followed by cranial nerve VI (41.3%), cranial nerve IV (30.4%), the ophthalmic branch of cranial nerve V (28.1%), and cranial nerve II (10.9%). Multiple nerves may be affected simultaneously in 54.3% of patients (33). The maxillary and mandibular divisions of the fifth nerve and even facial nerve may be affected (34, 35). Abnormal MRI findings occurr in only 52% of the patients (33). The patient presented here had involvement of the right cranial nerve III, IV, VI, and ophthalmic branch of V, as well as left cranial nerve VI and mandibular division of V. There was orbital pain behind the right eye. Other than the prolonged duration (3 weeks), the clinical picture would fit Tolosa–Hunt syndrome. MCC metastasis did not become the top differential diagnosis until MRI brain was obtained.

The patient was immunocompromised due to the use of immunosuppressants for his renal and pancreatic transplants. Immunosuppression is a significant risk factor, as the risk of developing MCC significantly increases with human immunodeficiency virus (36, 37), and chronic lymphocytic leukemia (38, 39). Solid organ transplant status has also been shown to increase the risk of developing MCC by 23.8-fold (40). In the study by Clarke et al., the incidence of MCC in renal transplant was 13.8 per 100,000 person-year, which translates to 25.7-fold increase in risk of developing MCC compared to the general population. In another study, the standardized incidence ratio of MCC is as high as 66 for patients with renal transplant (41). In that study, three cases of MCC were detected among 4,200 individuals who underwent renal transplantation. All three identified cases were on immunosuppressive medications. The latency between transplant and MCC ranged from 6 to 19 years. All three cases died and the survival time between diagnosis of MCC and death ranged from 0.5 to 2.1 years. Our patient had both renal and pancreatic transplant, which likely increased his risk of developing MCC. He developed MCC 8 years after his renal and pancreatic transplant, which is in line with the reported latency.

Merkel cell polyoma virus was detected in about 80% of all MCCs (42). Because immunosuppression has been shown to be a significant risk factor, it has been hypothesized that immunosuppression after an organ transplant increases the risk of Merkel cell polyoma virus infection and thus increases the risk of developing MCC. However, the study by Koljonen et al. (41) showed that only one out of the three MCC cases with renal transplant status (33%) was tested positive for Merkel cell polyoma virus. This rate was lower than the 80% expected in the general population. Even though these data argue against the hypothesis of polyoma virus being the underlying cause of MCC in organ transplant patients, the sample size is currently too small to draw a conclusion. Unfortunately, in the case presented here, we do not have the Merkel cell polyoma virus data available.

## Concluding Remarks

This case is unique because it is the only case report showing MCC metastasis to the clivus from a distant site. It also demonstrates that MCC metastasis symptoms can progress rapidly within weeks and can masquerade as symptoms of Tolosa–Hunt syndrome or cavernous sinus thrombosis.

## Ethics Statement

Retrospective case report (exempted). Informed consent was signed for publication. Patient was not identified.

## Author Contributions

KH drafted the manuscript and compiled the figures, tables, and videos. PD provided pathological images and reviewed the manuscript. MC designed and supervised the work and critically reviewed the manuscript.

## Conflict of Interest Statement

The authors declare that the research was conducted in the absence of any commercial or financial relationships that could be construed as a potential conflict of interest. The reviewer, AT, and handling editor declared their shared affiliation and the handling editor states that the process nevertheless met the standards of a fair and objective review.

## Supplementary Material

Video S1 Extraocular movements of the patient.
